# Case Report: Management of cerebral arterial gas embolism via transfer to an outpatient hyperbaric chamber

**DOI:** 10.3389/fmed.2025.1533459

**Published:** 2025-04-14

**Authors:** Emmanuel J. Thomas, Samuel J. Thomas, Jason A. Bailey, Jason M. Jaronik, Hassaan A. Khan, Manaal Buchh, Zenia Qasim, Saniya K. Zackariya, David E. Van Ryn, Mahmoud D. Al-Fadhl, Faisal Shariff, Hala K. Ansari, Kate M. Kelly, Ameera S. Khan, Jack H. Langford, Marcus Farrand, Eshaal Kizilbash, Reagan E. Ludwig, Jonathan Z. Zhao, Leigh K. Van Ryn, Caroline C. Howell, Marie Nour Karam, Anthony V. Thomas, Yunsheng Yan, Mark M. Walsh, Mathew K. Marsee

**Affiliations:** ^1^Department of Emergency Medicine, Saint Joseph Regional Medical Center, Mishawaka, IN, United States; ^2^Department of Emergency Medicine, Goshen Health, Goshen, IN, United States; ^3^Department of Emergency Medicine, Memorial Hospital, South Bend, IN, United States; ^4^Indiana University School of Medicine South Bend Campus, Notre Dame, IN, United States; ^5^Department of Medicine, University of Toledo Medical Center, Toledo, OH, United States; ^6^George Washington School of Medicine and Health Sciences, Washington, DC, United States; ^7^Department of Intensive Care Medicine, Women and Children’s Hospital of Chongqing Medical University, Chongqing, China

**Keywords:** gas embolism, hyperbaric oxygenation, large-core needle biopsy, iatrogenic disease, patient transfer, hospital outpatient clinics, emergency medical services, critical care

## Abstract

Gas embolisms can be caused by iatrogenic interventions, resulting in various manifestations. We present a patient who experienced loss of consciousness and simultaneous paralysis during a percutaneous needle biopsy of the lung. A CT scan of the head revealed a cerebral arterial gas embolism. Because the treating hospital did not have access to hyperbaric oxygen for immediate treatment, the patient was transferred to an outpatient wound care facility. There, the patient initially improved when treated with hyperbaric oxygen therapy but deteriorated with resumption of ambient pressure. Continued treatment occurred at another hospital where the patient’s condition normalized. The initial transfer of the patient to another facility was notable because it was a transfer from a rural hospital, a higher-level facility, to an offsite wound care center with a hyperbaric chamber, a lower-level facility that could provide a higher level of care. This case report demonstrates the importance of immediate treatment of iatrogenic gas embolism with hyperbaric oxygen, which often is not available at many hospitals, and highlights the necessity to adapt to the transport of the patient from a higher-level facility to a lower-level facility when such transportation is necessary to provide effective and immediate care. This report is not recommending routinely transferring such patients to a lower level of care facility. However, when deemed clinically necessary and safe by bedside emergency physicians/critical care pulmonary physicians, it is a viable option. Explicit guidelines for transfers to lower-level facilities should be established to avoid delays in these situations.

## 1 Introduction

**FIGURE 1:**
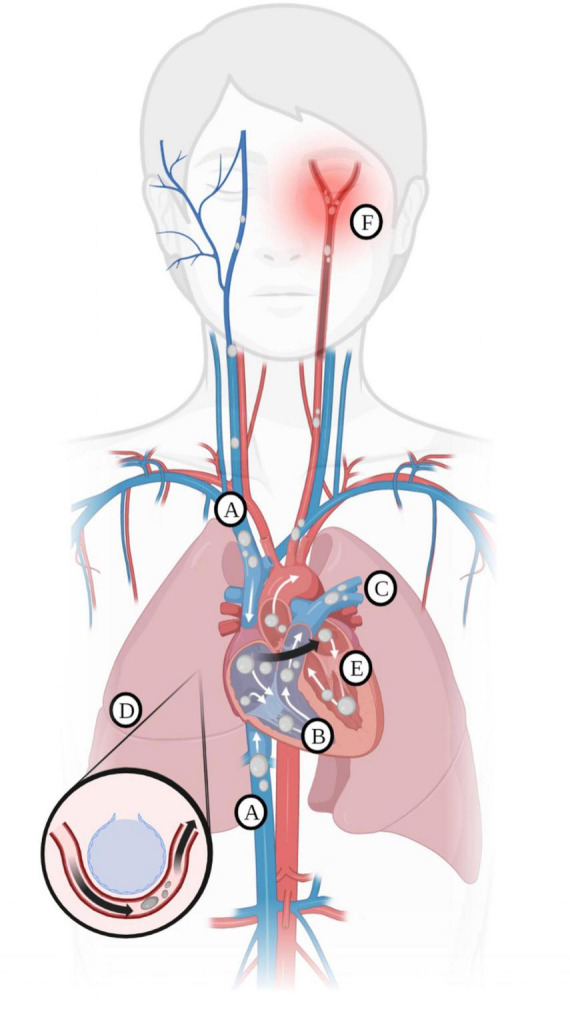
**(A)** Entry of gas into the superior and inferior vena cava. Most gas follows the superior or inferior vena cava into the right atrium. Less commonly, gas injected into the venous circulation will rise in a retrograde fashion into the cerebral circulation due to the natural buoyancy of the gas bubble and subsequently cause protean symptoms of venous occlusion of the cerebral vasculature. This scenario occurs, for example, with the injection of gas during the insertion or withdrawal of a central line in the sitting position. Note that due to the competing effects of buoyancy and drag, larger bubbles are more likely than smaller bubbles to become retrograde. **(B)** Gas in the right ventricle may totally occlude the pulmonary outflow tract, causing an “air lock” or “vapor lock” with resultant shock. **(C)** Gas may pass through the pulmonary artery and enter pulmonary circulation. **(D)** Gas may diffuse into the alveoli or be trapped in the pulmonary filter causing inflammation with impairment of gas exchange. **(E)** Gas may enter the left heart and systemic circulation directly by injection into the pulmonary vein or from the right heart via the lung, a right-to-left shunt, or a patent foramen ovale. **(F)** Systemic gas embolism, whether from the right heart or injected directly into the arterial circulation, causes end-organ damage, most commonly cerebral and cardiac. Reprinted with permission from Marsh et al. (12), licensed under CC BY 4.0. (Created with BioRender.com).

Gas embolism refers to an obstruction of blood flow caused by gas bubbles entering the vascular circulation (1). Iatrogenic gas embolism is an uncommon yet serious complication of many surgical procedures and operations, such as endoscopy, cesarean section, laparoscopic procedures, central venous access placement and removal, and others (2). Most clinically problematic iatrogenic gas embolisms occur when flow in small blood vessels is occluded by bubbles of gas that are accidentally injected into the bloodstream. Gas embolisms can affect both the arterial and venous circulations and can also cross over from venous to arterial circulation, a condition known as paradoxical embolism (3, 4). Figure 1 depicts the possible destinations of gas that enters the venous and arterial systems. This case report describes a cerebral arterial gas embolism (CAGE).

## 2 Case description

**FIGURE 2:**
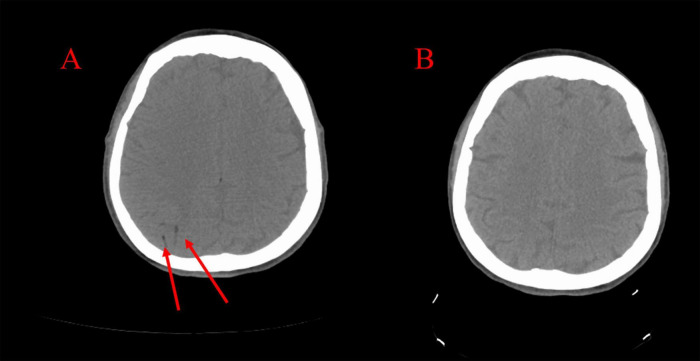
**(A)** CT scan taken immediately following the stroke demonstrating right-sided cerebral arterial gas embolism. **(B)** CT scan taken following hyperbaric oxygen therapy demonstrating resolution.

A 47-year-old woman suddenly lost consciousness and developed simultaneous-onset paralysis of the left arm and left leg during a CT-guided percutaneous needle biopsy of the right middle lobe of the lung to evaluate a hilar mass (biopsy was subsequently negative for malignancy). She had an eight-year history of peritoneal dialysis due to idiopathic renal failure and had undergone a nephrectomy for renal cell carcinoma several months prior to this presentation. In addition, the patient had a history of hypertension but no history of diabetes, thyroid disease, or bleeding disorders. The patient had no underlying risks for thromboembolism. Initial diagnoses that were considered were related to possibility of neurotoxicity caused by therapeutic opioids and benzodiazepine, and therefore, intravenous naloxone and flumazenil were administered for unresponsiveness, which had no effect. Then because of the focal deficit associated with unresponsiveness, the interventional radiologist suspected arterial gas embolism and initiated 100% oxygen via a non-rebreather mask. The biopsy procedure was immediately discontinued, the needle was removed, and the biopsy tract was sealed with a BioSentry tract sealing device (AngioDynamics, Latham, NY, United States). A CT of the chest confirmed the absence of any pneumothorax. Because the patient was already in the CT scanner for the procedure, a non-contrast head CT was immediately obtained and revealed CAGE (Figure 2A). The patient was transferred to the emergency department for further management. She regained consciousness but had sustained left hemiparesis. Attempts to transfer the patient to a facility with an inpatient critical care-capable hyperbaric unit were unsuccessful; five contacts were made with academic and nonacademic centers with multiplace hyperbaric chambers capable of accommodating a respiratory therapist and a ventilator, and they all declined this patient due to lack of staff and resource availability. The search radius extended 215 miles. It was believed that the likelihood of finding a hyperbaric chamber facility within the next 24 h was unlikely. Lacking other options, a decision was made to initiate hyperbaric oxygen therapy (HBOT) at a freestanding outpatient wound care center. This transfer was deemed appropriate because the patient was awake, alert, and protecting her airway and thus low risk for clinical deterioration while inside the chamber. Thus, the patient was transferred by the local fire department while on 100% oxygen. While undergoing HBOT at the outpatient facility, the patient had physician and paramedic supervision. The paramedics and physicians were able to administer, if needed, advanced cardiac life support (ACLS) measures including mechanical ventilation.

The patient was treated in a Sechrist 3200R monoplace hyperbaric chamber according to Treatment Table 6 of the United States Navy Diving Manual, Revision 7. This table outlines the recommended HBOT protocols for diving-related arterial gas embolism and is used for treating iatrogenic gas embolism as well. The patient was treated at an initial pressure of 3 atmospheres absolute (ATA) for approximately 4 h while breathing 100% oxygen and was visually monitored for any developments. Since there was no room in the chamber for instruments for cardiac or oxygen monitoring, there were scheduled air pauses to reduce the risk of oxygen toxicity. After the initial treatment, the pressure was gradually reduced according to a specific decompression schedule while continuing oxygen therapy. If symptoms persist or return after initial treatment, additional sessions using the same or modified tables may be recommended. Treatment Table 6 emphasizes prompt treatment to reduce the risk of permanent damage from gas embolism (5). There are alternative strategies that have been suggested to treat CAGE in the absence of hyperbaric oxygen, but the use of positive end expiratory pressure with mechanical ventilation and extracorporeal membrane oxygenation were not considered because this patient did not require intubation.

The patient returned to normal consciousness and had immediate resolution of her left-sided weakness upon reaching 3 ATA at the initiation of HBOT. However, upon returning to 1 ATA at the conclusion of the treatment protocol (8 h post-biopsy), she developed decorticate posturing and had diminished consciousness.

The patient was taken out of the hyperbaric chamber and emergently returned to the local emergency department where she was evaluated by 3 emergency physicians. Her Glasgow Coma Scale (GCS) was 10 [eye opening 4 (E4), verbal response 3 (V3), motor response 3 (M3)]. She continued to protect her airway and maintained a gag reflex, so she was not intubated. It was deemed necessary that she be transferred to a critical care facility with hyperbaric chamber capacity so that she could undergo further HBOT. Patient transport was supervised by experienced paramedics capable of endotracheal intubation in case of further decompensation.

Upon arrival at the accepting hospital, the patient had maintained a GCS of 10 and was still protecting her airway. A critical care physician was consulted and did not recommend endotracheal intubation. The patient underwent repeat CT scanning of the head, which showed resolution of the CAGE (Figure 2B). On the evening of the biopsy, she was noted to have a flexor response of the upper extremities and lower extremities with hyperreflexia of the brachioradialis, patellar, and Achilles tendons, and four-beat ankle clonus. Her GCS score measured a total of 9 (E4, V2, M3) indicating a fluctuating level of consciousness, yet she continued to protect her airway. The laboratory data were within normal limits. A repeat CT scan with angiography of the head and neck revealed no further visible bubbles in the brain, and all vessels appear patent.

**FIGURE 3:**
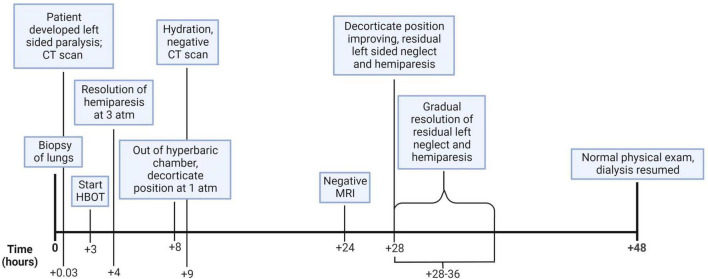
This figure illustrates the hourly timeline of the clinical course and medical decision making. (Created with BioRender.com).

Neurology and critical care consultants recommended delaying peritoneal dialysis to hemodilute the patient and improve local blood flow around the bubbles. Since this patient’s renal failure and reliance on dialysis complicated fluid management and because the patient had not received much fluid during the day of the procedure and subsequent admission to the previous hospital, it was elected to not provide hemodialysis because she appeared euvolemic. It is known from study of the hemodynamics and pathophysiology of the effect of bubbles on vascular blood flow in the brain and other structures that judicious use of intravenous fluids and maintaining a physiologic central venous pressure provides the best milieu for the dissolution of bubbles during HBOT (1, 2). The patient was maintained on 100% oxygen by non-rebreather mask. The next morning, she underwent an MRI of the head which showed no abnormality. Later that day, approximately 28 h after the onset of focal weakness and approximately 20 h after the onset of decorticate posturing, the patient gradually awakened with a GCS of 13 (E4, V5, M4). 35 h after the biopsy, the hyperreflexia and muscle hypertonicity diminished, and the patient had left-sided palsy and weakness of the arm and leg. An electroencephalogram taken 30 h after the biopsy was abnormal due to the presence of moderate, diffuse slowing of the background rhythms and superimposed right hemispheric theta delta slow activity without epileptiform features, which are indicative of superimposed right hemispheric dysfunction. On the second day, the patient resumed hemodialysis due to symptom improvement. During this entire time, the patient received 100% oxygen through a non-rebreather mask. Due to the patient’s improvement in signs/symptoms and the absence of cerebral gas on the repeat CT scan, it was decided not to pursue further HBOT. Over the next two days, the patient’s condition returned to normal with complete resolution of paralysis. Four days after admission, the neurologic exam was completely normal, and she was discharged (Figure 3). Two years later, the patient is free of symptoms.

## 3 Discussion

**FIGURE 4:**
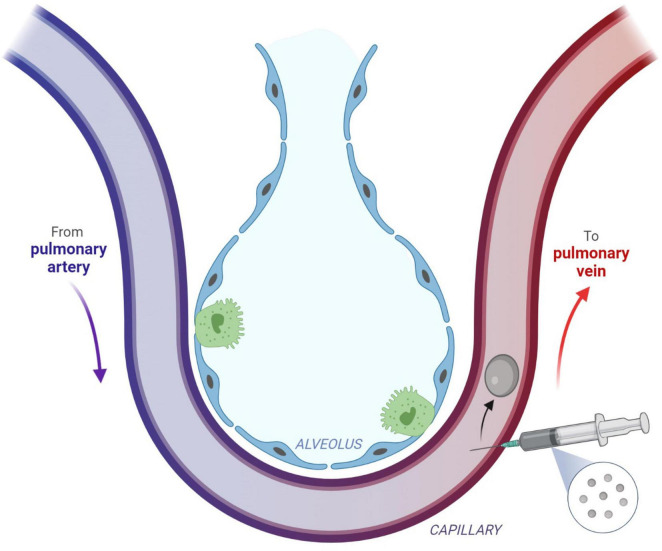
Direct gas embolism is caused by the entry of gas into the pulmonary veins or directly into the arteries of the systemic circulation. This figure describes the injection of gas into the pulmonary venous circulation, which can occur after a procedure such as a biopsy. Subsequent arterial gas embolism can occur immediately after the biopsy. Reprinted with permission from Marsh et al. (12), licensed under CC BY 4.0. (Created with BioRender.com).

Systemic gas embolism following a percutaneous core needle biopsy of the lung has been presented in the literature as an uncommon, yet potentially severe complication of this procedure (6–11). If this complication occurs, HBOT is the standard treatment (12). Figure 4 shows the mechanism of gas entry into the pulmonary venous circulation system during the lung biopsy.

In our case, the patient rapidly improved within minutes of reaching 3 ATA via HBOT, which likely correlated with a reduction of bubble size in the middle cerebral artery territory (12, 13). The patient’s subsequent deterioration with decorticate posturing at ambient pressure was presumably due to the appearance of smaller bubbles diffusing through the cerebrum, resulting in endothelial inflammation and a low flow hibernating cerebral cortex with global penumbral blood flow (12, 13). The recurrence of symptoms once the patient returned to atmospheric pressure followed the description in existing literature concerning the delay in treatment for patients who incurred symptoms of decompression sickness (DCS) following diving sessions and experienced persistent or worsening symptoms upon flying home (13, 14). There is an increased need for observation units for those who have received a single treatment, since recurrence has been noted after these patients are treated at 3 ATA and returned to ambient pressure (12, 13), as in this case. For patients with diving-associated DCS, which usually occurs during vacations in warm water coastal areas, it is not uncommon for a patient to receive HBOT. It is well known that following an improvement of symptoms with HBOT, there may be a delayed recurrence of symptoms due to the return of gas bubbles into the circulation and tissue (15). For example, a patient with DCS receives one or two treatments in an outpatient facility at the vacation site in the warm weather venue, only to worsen with a recurrence of symptoms caused by the lower pressure of high altitude during the flight home. In their community, however, there may be a number of outpatient hyperbaric chambers which could accommodate these traveling patients who arrive home and present to an emergency department, a higher level of care facility which lacks access to HBOT. The recent promulgation of outpatient monoplace hyperbaric chambers has allowed for the evolution of the treatment of patients, including acutely ill hospitalized patients, in outpatient facilities affiliated with the main hospital in a setting away from the main hospital campus (15).

The risk of a patient deteriorating while in a hyperbaric chamber, either from a respiratory or cardiac arrest, needs to be considered before the patient is placed in that chamber. Patients should be carefully selected for HBOT, weighing the risk of cardiorespiratory decompensation with the benefit of therapy (5). For this patient, it was determined that the benefits of treatment outweighed the risks, and it was decided to proceed with HBOT. She was monitored throughout the course of therapy for signs of clinical deterioration and cardiorespiratory decompensation and never required intubation or cardiopulmonary resuscitation. There are no guidelines for patients who undergo a cardiorespiratory decompensation during HBOT. Recent attempts to establish guidelines have suggested organizing drills of this scenario simulating cardiorespiratory decompensation during HBOT in a monoplace hyperbaric chamber (16). As the maximum rate of decompression varies from chamber to chamber, each HBOT facility should have its own emergency decompression protocol per the specifications of its device. For example, the maximum emergency depressurization time from 3 ATA to 1 ATA for a Sigma 40 monoplace hyperbaric chamber is 120 seconds (17). This protocol should balance the risk of DCS with the urgency of the need to provide cardiac and airway support. HBOT facilities should also have infusion pumps to allow the administration of medications during treatment and be staffed with personnel capable of administering ACLS treatment.

Many situations require the transfer of a patient from a lower level of care to a higher level of care when the lower-level facility cannot adequately treat the patient. This allows for the patient’s treatment to be managed by more specialized and experienced providers with access to the specific necessary medical equipment. Any delays in patient transfer can lead to deterioration of patient status and diminished effectiveness of specialized care (18).

This case represents a unique and informative example of a successful transfer of a patient from a higher-level to lower-level facility to provide a greater level of care. Approximately three hours were spent contacting higher-level care facilities with inpatient critical care-capable multiplace hyperbaric units, and none had staff and resource availability to accommodate this patient. Therefore, the patient was transferred from a hospital to an offsite wound care center with a hyperbaric oxygen chamber. Because the patient’s GCS was 10 and she was protecting her airway, she was able to be transferred. Had this patient not protected her airway and had a GCS of 8 or below, she would have required endotracheal intubation, and the transfer would not have been possible. This case raises the question of whether stable, less critically ill patients could be transferred from hospitals without hyperbaric oxygen chambers to outpatient facilities for HBOT with subsequent return to the originating hospital for reassessment, rather than necessitating long-distance transfers to hospitals with both critical care and hyperbaric capabilities.

European literature has described a formal arrangement that allows for patients hospitalized in an intensive care unit to be transferred to a single freestanding monoplace hyperbaric chamber facility for therapy (18). This scenario allows one to probe the legal history of the United States, which has established the American College of Emergency Physicians (ACEP) transfer guidelines and the Emergency Medical Treatment and Labor Act (EMTALA) to provide direction regarding patients who require medical services not available at the institutions where they are hospitalized (19, 20). International guidelines reflect the same intent to provide clear-cut rules for transferring patients from lower to higher levels of care and are in line with the ACEP and EMTALA guidelines (18, 21–32).

Although the ACEP and EMTALA documents describe the transfer from a lower to higher level of care, the transfer of critically ill patients from a higher-level to lower-level facility where unique medical, diagnostic, and therapeutic modalities are available (such as outpatient hyperbaric chambers) can be tacitly supported via interpretation of the ACEP guidelines. Given the first principle listed in the ACEP guidelines (“The optimal health and wellbeing of the patient should be the principal goal of the transfer”) and the ensuing principles listed including the consideration of equipment needed for care, we believe that the ACEP guidelines allow the transfer of a patient from a higher- to lower-level facility for necessary specialized equipment and therapy (19). EMTALA is designed to prevent discrimination against patients without insurance. Among the legal requirements of EMTALA is that for patients who have an emergency medical condition that cannot be treated at the hospital requesting transfer, a hospital with specialized services able to treat the patient must accept the patient if bed availability exists. EMTALA legislation does not account for the transfer of a patient from a higher to lower level of care facility if the higher-level institution does not have the specialized facilities to treat the patient (20). In any event, any such decision to conduct a transfer of this nature should be made only after a bedside emergency/critical care physician establishes the safety of the patient for transfer.

Therefore, the treatment team interpreted these guidelines in such a way to justify transfer of a stable patient from a higher-level institution where there was not specialized HBOT equipment to a lower-level institution where the facilities existed. Because of preexisting inter-institution relationships, this transfer was accomplished with minimal delay and did not require administrative approval. However, the process of interpretation that allows for this type of transfer from a hospital to a freestanding outpatient hyperbaric chamber facility may be difficult and inefficient in the acute moments when the patient needs immediate transfer. Even with strong existing inter-facility relationships and guidelines in place, considerable modulation and administrative improvisation may be required of hospital administrators and the EMS director and personnel in arranging for timely transfer of the patient. It is for this reason that written agreements between outpatient facilities and hospitals should be implemented to facilitate a seamless transfer during acute illness. Based on the last principle listed in the ACEP guidelines (“When transfer of patients is part of a regional plan to provide optimal care at a specialized medical facility, written transfer protocols and interfacility agreements should be in place”) (19), we recommend emergency department care teams consider, and to the best of their ability predict and prepare for, any such need for these unusual transfers and update the relevant regional plans to ensure appropriate care can be promptly rendered in such inevitably time-critical situations. As a foundation for guiding future study into the developing formal guidelines and protocols for transferring patients from a higher to lower-level facility, modifications of such guidelines as the ACEP policies should highlight the importance of the judgment of the individual physician regarding the appropriateness of transfer. Emphasis should be placed on the fact that the current ACEP guidelines suggest the use of physician judgment regarding destination of patient transfer (19).

The authors consider it serendipitous that the higher-level facility was a rural hospital that had close connections to a local freestanding hyperbaric chamber facility, which allowed for the unusual but lifesaving transfer of the critically ill patient to receive HBOT. An inevitable limitation is that this remains the first case report of an iatrogenic CAGE treated after a transfer to a lower-level outpatient facility for HBOT. However, the European literature has described transfer of critically ill, mechanically ventilated patients with a variety of severe illnesses such as necrotizing fasciitis and sepsis to a standalone HBOT facility (18). We conclude this case report with a reminder to practitioners that transfer from a nominally higher-level to a lower-level facility is not forbidden despite the common presupposition that such a prohibition exists.

## Data Availability

The original contributions presented in the study are included in the article/supplementary material, further inquiries can be directed to the corresponding author.
